# Placental Stromal Cell Therapy for Experimental Autoimmune Encephalomyelitis: The Role of Route of Cell Delivery

**DOI:** 10.5966/sctm.2015-0363

**Published:** 2016-09-29

**Authors:** Ilona Shapira, Nina Fainstein, Maria Tsirlin, Ilana Stav, Evgenia Volinsky, Claudia Moresi, Tamir Ben‐Hur, Raphael Gorodetsky

**Affiliations:** ^1^ Department of Neurology, The Agnes Ginges Center for Human Neurogenetics, Hadassah Hebrew University Medical Center, Jerusalem, Israel; ^2^ Laboratory of Radiobiology and Biotechnology, Sharett Institute of Oncology, Hadassah Hebrew University Medical Center, Jerusalem, Israel

**Keywords:** Cell therapy, Multiple sclerosis, Placenta, Route of cell delivery

## Abstract

Multiple sclerosis (MS) is an immune‐mediated disease of the central nervous system (CNS) with no effective treatment available for the chronic‐progressive stage. Cell therapy is a promising therapeutic approach for attenuating the immune‐mediated CNS process. Isolated and expanded human placental stromal cells (hPSCs) possess potent immunomodulatory and trophic properties, making them a good candidate for MS therapy. We examined the potential of hPSC therapy in preventing the onset or attenuating the course of established disease in a murine MS model of myelin oligodendrocyte glycoprotein‐induced experimental autoimmune encephalomyelitis. We examined the feasibility of hPSC systemic delivery by intramuscular (i.m.) implantation rather than the commonly used intravenous injection, which is dose‐limiting and carries the risk of pulmonary obstruction. Our findings showed significant attenuation of the disease only when hPSCs were injected directly to the central nervous system. Intramuscular implanted hPSCs survived at the site of injection for at least 2 months and elicited extensive local immune responses. Intramuscular hPSC implantation before disease onset caused a delay in the appearance of clinical signs and reduced the severity of a relapse induced by repeated challenge with the autoantigen. Intramuscular implantation after disease onset did not affect its course. Thus, pathological analysis of CNS tissue did not show inhibition of neuroinflammation in i.m. hPSC‐implanted mice. Moreover, no apparent effect was seen on the proliferative response of peripheral lymph node cells in these animals. We conclude that to maximize their therapeutic potential in MS, hPSCs should be delivered directly to the affected CNS. Stem Cells Translational Medicine *2017;6:1286–1294*

**Significance Statement**

Emerging cell therapy approaches for multiple sclerosis (MS) use their immunomodulatory and neurotrophic properties to ameliorate neuroinflammation and facilitate repair processes. The central unsolved issues include the choice of the optimal cell platform and the preferred route of cell delivery. The results of the present study show that placental cells are effective in attenuating experimental autoimmune encephalomyelitis, the animal model of MS, when delivered directly to the central nervous system (CNS). However, peripheral intramuscular delivery was associated with a strong local immune reaction and a limited clinical therapeutic effect. This study’s results support direct cell delivery to the CNS.

## Introduction

Multiple sclerosis (MS) is an immune‐mediated, multifocal, chronic disease of the central nervous system (CNS). The disease is characterized by perivascular immune cell infiltrates, myelin destruction, and axonal loss, leading to permanent neurological disability. Cell therapy supported by the implantation of different cell types has been proposed as a potential therapy for regenerative medicine in progressive MS [1, 2, 3, 4, 5, 6, 7, 8, 9, 10]. No effective treatments are available for this stage of disease; hence, a major need exists for the development of novel therapies. Multiple studies have underscored the immunomodulatory and neurotrophic bystander effects shared by various types of stromal/stem cells. Specifically, both neural stem/precursor cells and mesenchymal stromal cells (MSCs) were shown to ameliorate experimental autoimmune encephalomyelitis (EAE), one of the most practiced animal models of MS [1, 2, 4, 6, 9]. These functional properties have become the main rationale for cell therapy in MS with current clinical experimentation. Crucial issues that are still under debate concern the choices of optimal cell platforms and the optimal route of cell delivery [10, 11].

Recent studies have also suggested that stromal cells isolated and expanded from different layers of the human placenta (human placental stromal cells [hPSCs]) might represent a valid option for cell therapy with significant immunomodulatory properties [12, 13, 14, 15]. This cellular platform is both ethically accepted and easily expandable to produce large batches of an off‐the‐shelf cell product for allogeneic implantation.

Several approaches are available supporting different routes of cell delivery to achieve the desired effect. A major rationale for an intracerebroventricular (ICV) and/or intrathecal route of cell delivery is that implanted cells disseminate in the CNS ventricular/subarachnoid space in close proximity to most white matter tracts that are involved in MS and migrate efficiently into the tissue. Alternatively, systemic cell delivery could be considered. Intravenous (i.v.) delivery of several types of adult stromal cells caused systemic immunomodulation, albeit with a lack of profound cell entry into the brain parenchyma. The delivered cell doses are limited because of the well‐documented findings that most i.v. delivered cells are immediately trapped in the lungs after injection [16, 17, 18], causing transient severe stress and a significant increase in the risk of pulmonary thrombosis. This might also shorten the duration of their paracrine effect [16]. As an alternative to i.v. cell delivery, the possibility of using intramuscular (i.m.) implantation of hPSCs was suggested. The cells implanted into the highly vascularized muscle mass can be adequately nourished while exposing the cells to systemically carried factors and allowing the delivery of their secreted factors into the circulation. This approach of injecting into the muscle mass allows at least a doubling of the number of delivered cells per injection compared with i.v. injection, with no apparent adverse effects and with prolonged residence of the cells in the injected site [16]. The ability to increase the dose of delivered cells could be advantageous given the dose‐dependent nature of the immunomodulatory effect of stem cells [2]. A recent study showed that i.m. delivery of a similar preparation of mixed fetal and maternal hPSCs efficiently mitigated the effect of lethal radiation syndrome to enable a lifesaving fast recovery of the affected bone marrow [16]. Therefore, we studied the therapeutic potential of hPSCs in EAE and whether i.m. cell implantation is an adequate method of cell delivery.

Our results suggest that although ICV injection of hPSCs to EAE mice causes a significant delay in disease onset and attenuation of clinical severity, i.m. injection caused only a mild delay in EAE onset with limited improvement in the severity of the disease. These results were indicated by both the clinical signs and the findings of the histopathological evaluation. Thus, our results support the therapeutic approach of hPSC delivery directly to the target organ, the CNS.

## Materials and Methods

### Animals

Female C57Bl/6 mice, 6–7 weeks old, were supplied by Harlan Laboratories (Indianapolis, IN, http://www.envigo.com) and kept under specific pathogen‐free conditions. The Hadassah Hebrew University Medical School’s ethics committee approved the animal experimentation.

### Isolation, Growth, and Characterization of hPSCs

**Figure 1:**
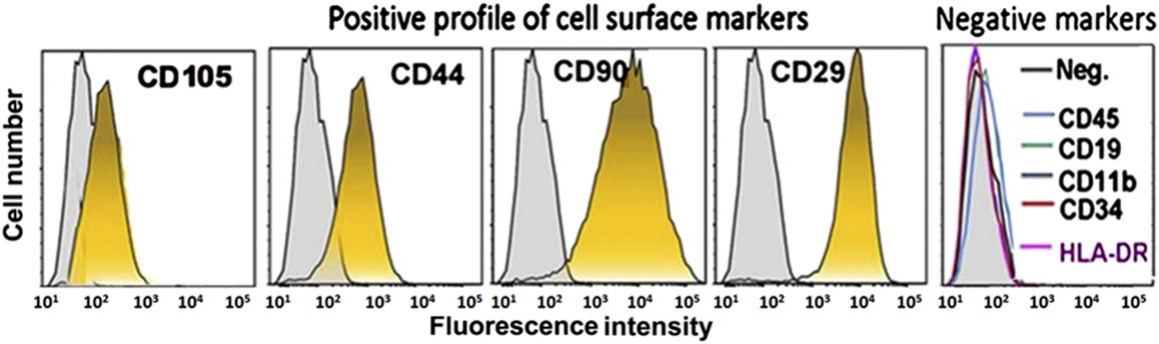
Characterization of isolated human placental stromal cells as stromal cells by cell surface marker fluorescence‐activated cell sorting analyses. The cells were negative for markers of hematopoietic or endothelial progenies (CD45, CD19, CD11b, HLA‐DR, and CD34) and positive for typical mesenchymal stromal markers (CD105, CD44, CD29, and CD90). Abbreviations: HLA, human leukocyte antigen; Neg., negative.

A placenta was obtained in sterile conditions immediately after cesarian section of a healthy male child by a healthy mother, with her written consent. The placenta was then rinsed extensively, and small fractions from the surface decidua of the chorion in the fetal side were cut into small pieces, which were then further minced to smaller tissue fragments. These were briefly digested twice with trypsin immersed in medium with highly diluted (1%) fibrinogen (Omrix Biopharmaceuticals, New York, NY, http://www.ethicon.com) and then distributed on a plastic dish and sprayed with thrombin (100 U/ml) to adhere them to the plastic surface of the culture dish. The pieces were then cultured with low‐glucose Dulbecco’s modified Eagle’s medium with 10% fetal calf serum, 1% antibiotics (penicillin/streptomycin, optionally with neomycin), and 1% glutamine (all from Biological Industries, Beit Haemek, Israel, http://www.bioind.com). The placental stromal cells from these selected tissue fragments spontaneously migrated to the plastic surface to reach a stable uniform culture of pure stromal cells (CD90+, CD29+, CD105+ partially positive and CD11b, CD34, CD45, and HLA‐DR negative; Fig. 1). The cells had very low human leukocyte antigen (HLA)‐DR expression. Fish staining of the X/Y centromeres enables the identification of the source of the cell from the placenta, the mother’s being X/X and the newborn’s X/Y. Cultures of isolated cells in which most were from the fetal source (>80%) were used for the experiments. The cells were then expanded for five to eight passages before their injection. They were harvested for implantation and counted, rinsed, and resuspended in PlasmaLyte A (Baxter International, Deerfield, IL, http://www.baxter.com). Cell preparation with a viability of <90% was injected i.m. within 1–2 hours from their harvest.

### Experimental Induction of Autoimmune Encephalomyelitis With Intracranial or Intramuscular hPSC Delivery

Mice were immunized by subcutaneous injection of 300 μg of myelin oligodendrocyte glycoprotein (MOG) peptide corresponding to amino acids 35–55 (MOG35–55), diluted in normal saline (0.9% NaCl), and emulsified with complete Freund's adjuvant. *Bordetella pertussis* toxin (300 ng; List Biological Laboratories, Inc., Campbell, CA, http://www.listlabs.com) was diluted in 0.2 ml of normal saline and injected i.p. immediately after MOG immunization and 2 days later. Clinical signs of EAE typically appeared 10–12 days after immunization, reaching the peak neurological disability within an additional 6–10 days. After induction, 85% of the mice developed the disease. Disease progression and severity was scored daily as follows: 0 = normal; 1 = limp tail; 2 = ataxia; 3 = partial hind limb weakness and/or an inability to flip over; and 4 = hind limb scored independently by three researchers in turn in a nonblinded manner. The data were analyzed by two of the three at the end of the experiment. For relapse induction, the mice were immunized again with the MOG peptide and *B. pertussis* toxin, starting on day 40, as previously described [19].

Intramuscular implantation to the quadriceps and hamstrings in total amount of 2 × 10^6^ hPSCs in 100 µl of PlasmaLyte A was performed on days 0 and 5 after EAE induction to study the preventive effect and on days 11 and 15 after EAE induction to study the treatment protocol. Intracranial stereotactic implantation (bregma, 0 mm; lateral, 0.5 mm) of 0.5 × 10^6^ hPSCs in 10 µl of PlasmaLyte A was performed on day 7 after EAE induction. The control mice were followed up without intervention. The mice were anesthetized for the invasive procedures with ketamine/xylazine [20]. The mice were scored daily for neurological symptoms and perfused on day 28 or 60 for histopathological analysis [19].

### Lymphocyte Isolation, In Vitro Proliferation Assay, and Regulatory T‐Cell Staining

Lymph node cells (LNCs) were excised from EAE‐induced mice at 7 or 21 days after MOG immunization. LNCs were cultured in 24‐well plates (1 × 10^6^ cells per well) with Roswell Park Memorial Institute medium supplemented with 10% fetal calf serum, 1 mM l‐glutamine, and antibiotics. The LNCs were stimulated with 120 µg/ml MOG35–55 or 2.5 µg/ml concanavalin A (ConA). To evaluate LNC proliferation, 2 mM bromodeoxyuridine (BrdU) was added for 1 hour after 48 hours of incubation in a humidified atmosphere of 55% CO_2_ at 37°C. To assess the percentage of regulatory T cells, LNCs were immediately stained for CD4, CD25, and Foxp3 (regulatory T‐cell kit; eBioscience, San Diego, CA, http://www.ebioscience.com). Analysis was performed using fluorescence‐activated cell sorting (FACS) (BD Biosciences, San Jose, CA, http://www.bdbiosciences.com).

### Lymphocyte Isolation From Brain and Spinal Cord

Using the Percoll (GE Healthcare, Port Washington, NY, http://www.gehealthcare.com) procedure, lymphocytes were excised from the brains and spinal cords of EAE‐induced mice 22 days after MOG immunization. The cells were precoated with anti‐mouse CD16/CD32 (BD Biosciences) to block unspecific binding and stained with anti‐Thy1.2 for T cells, CD11b for macrophages/microglia (BD Biosciences), and, alternatively, activated macrophages by double staining of CD11b and CD206 (BD Biosciences).

### Histopathologic Examination

The mice were anesthetized with a lethal dose of pentobarbital and perfused via the ascending aorta with ice‐cold phosphate‐buffered saline (PBS), followed by cold 4% paraformaldehyde in PBS. The brains, spinal cords, and muscles were deep frozen in dry ice, serial 10‐μM sections were made, and immunofluorescent staining for T cells (anti‐CD3; AbD Serotec, Kidlington, UK, http://www.abdserotec.com), macrophages/microglia (anti‐Iba1; Wako Pure Chemical Industries, Ltd., www.wako-chem.co.jp), M1 macrophages (anti‐inducible nitric oxide synthase [iNOS]; EMD Millipore, Billerica, MA, http://www.emdmillipore.com), anti‐M2 macrophages (anti‐arginase; EMD Millipore), and hPSCs (anti‐human HLA; Acris, OriGene Technologies Inc., Rockville, MD, http://www.origene.com) were performed as previously described [19]. T cells were counted manually from five microscopic fields (at ×40 magnification) for each section of spinal cord per mouse. The average number of cells was calculated for each mouse, followed by calculating the group average ± SEM. Iba1+ cells were quantified from five microscopic fields (at ×40 objective magnification) for each section of spinal cord using a computer software program counting pixels. The number of infiltrates was quantified on histological sections throughout the entire spinal cord using the 4′,6‐diamidino‐2‐phenylindole nuclei counterstain.

### Statistical Analysis

The outcome variable in all clinical comparisons between the treated and control groups was the cumulative clinical score. The shape of the distribution of the score and the number of observations in each experiment did not justify the assumption of normality needed to use linear models (e.g., *t* test or analysis of variance). Consequently, the nonparametric Mann‐Whitney *U* test was used. All *p* values reported are two‐tailed.

The pathological quantifications and FACS analysis in the transplantation experiments between the groups of control mice and hPSC‐transplanted mice were calculated using Student's independent (unpaired) samples *t* test. The probability of developing a relapse was compared between the groups using the chi‐square test.

## Results

### Characterization of hPSCs

The surface markers of the cultured hPSCs were assayed using FACS analysis (FACSCalibur; BD Biosciences), verifying negative hematopoietic lineage markers (CD45, CD19, CD11b, CD34, HLA‐DR) and positive expression of CD105, CD44, CD90, and CD29 (antibodies purchased from PeproTech, Rocky Hill, NJ, http://www.peprotech.com).

### Intracranial Implantation of hPSCs Attenuates EAE

**Figure 2:**
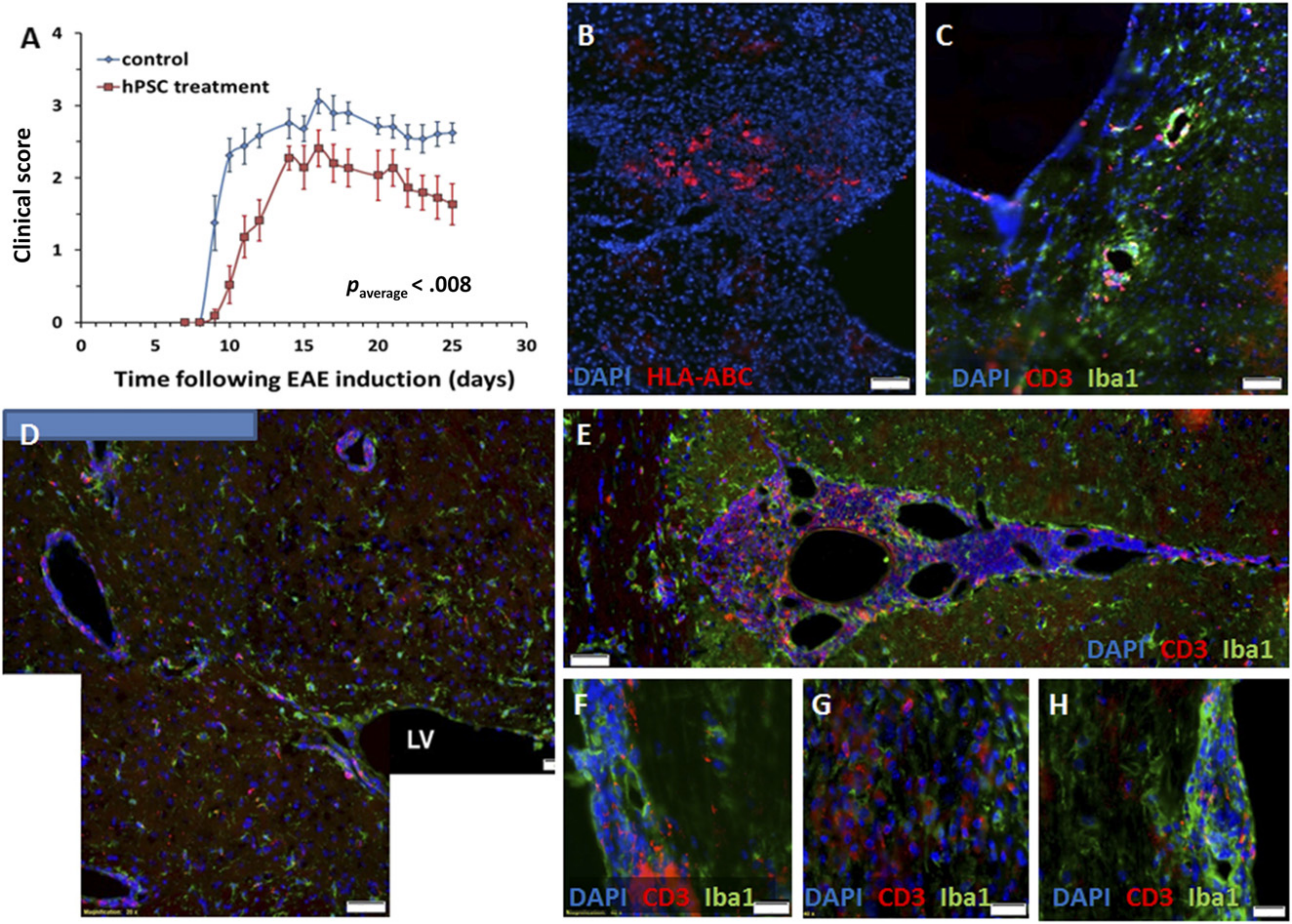
Intracranial implantation of hPSCs to EAE mice. hPSCs were implanted in the periventricular white matter of mice on day 7 after EAE induction before the development of clinical signs (*n* = 12 mice). **(A):** Significant (*p* = .001) disease attenuation was observed in the clinical course of hPSC‐implanted mice compared with a control EAE group (*n* = 11). **(B):** hPSCs were detected 18 days after implantation by HLA‐ABC immunostaining of the mouse brain sections. **(C):** EAE mice exhibited a typical inflammatory response in their brains on day 25 after EAE induction. **(D, E):** A strong inflammatory response was noted in the hPSC‐transplanted mice in proximity to the implantation site and in the brain meninges. Both meningeal and parenchymal spinal cord infiltrates were detected in the EAE mice **(F, G)**, but the hPSC‐implanted mice exhibited mostly meningeal infiltrates **(H)**. Scale bars = 50 µm **(B–E)** and 20 µm **(F–H)**. Abbreviations: DAPI, 4′,6‐diamidino‐2‐phenylindole; EAE, experimental autoimmune encephalomyelitis; HLA, human leukocyte antigen; hPSC, human placental stromal cell; LV, lateral ventricle.

To examine whether hPSCs exhibited effective immunomodulatory properties in the animal model of MS, we first performed intracranial implantation of hPSCs to the periventricular white matter tracts of EAE mice (*n* = 11 and 12 per group). hPSCs were implanted on day 7 after EAE induction, before the appearance of clinical signs. Significant disease attenuation was observed in the hPSC‐implanted EAE mice compared with the nonimplanted mice (*p* = .001; Fig. 2A). The mice were sacrificed for histopathological evaluation at 25 days after EAE induction (18 days after cell implantation). The hPSCs were detected by immunostaining for HLA‐ABC at the site of implantation in the mouse brains (Fig. 2B) and in the ventricles. A typical inflammatory process was found in the spinal cords and brains of the EAE mice (Fig. 2C). In the cell‐implanted mice, a strong local inflammatory response was noted around the site of implantation and in the brain meninges (Fig. 2D, 2E). The number of inflammatory infiltrates was quantified throughout the spinal cord; 4.42 ± 1.18 infiltrates per section were found in the control mice compared with 4.02 ± 0.64 infiltrates per section in the hPSC‐treated mice (*p* = N.S.). In the control mice, the inflammatory process also involved the spinal cord parenchyma (Fig. 2F, 2G). However, in the hPSC‐treated mice, it was almost exclusively meningeal (Fig. 2H). These differences might reflect the meningeal immune response toward the graft itself.

### Implanted hPSCs Can Persist in the Muscle for at Least 60 Days

**Figure 3:**
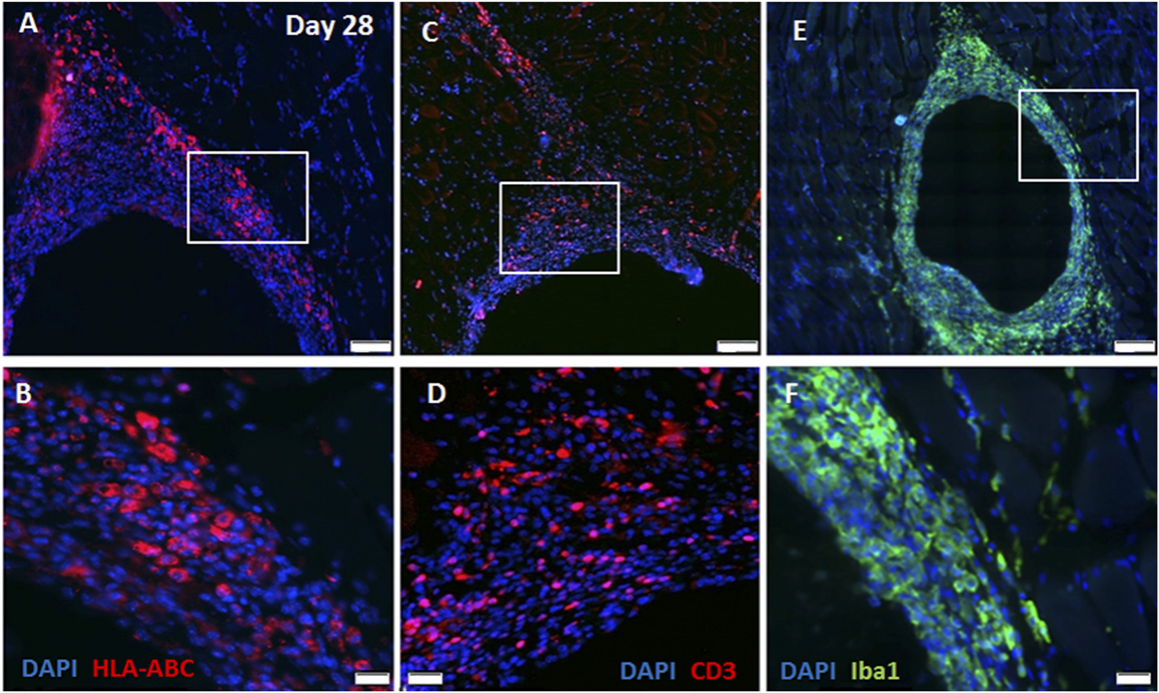
Intramuscularly implanted human placental stromal cells (hPSCs) survive and elicit an immune response. At 28 days after intramuscular implantation, numerous hPSCs were detected in the recipient muscles by HLA‐ABC immunostaining **(A, B)**. A significant immune response was evident in proximity to the graft, as manifested by extensive infiltration of CD3^+^ T cells **(C, D)** and Iba1+ macrophages **(E, F)**. Insets in **(A, C, E)** are magnified in **(B, D, F)**, respectively; *n* = 5) in **(C)**. Scale bars = 100 µm **(A–C)** and 20 µm **(D–F)**. Abbreviations: DAPI, 4′,6‐diamidino‐2‐phenylindole; HLA, human leukocyte antigen.

**Figure 4:**
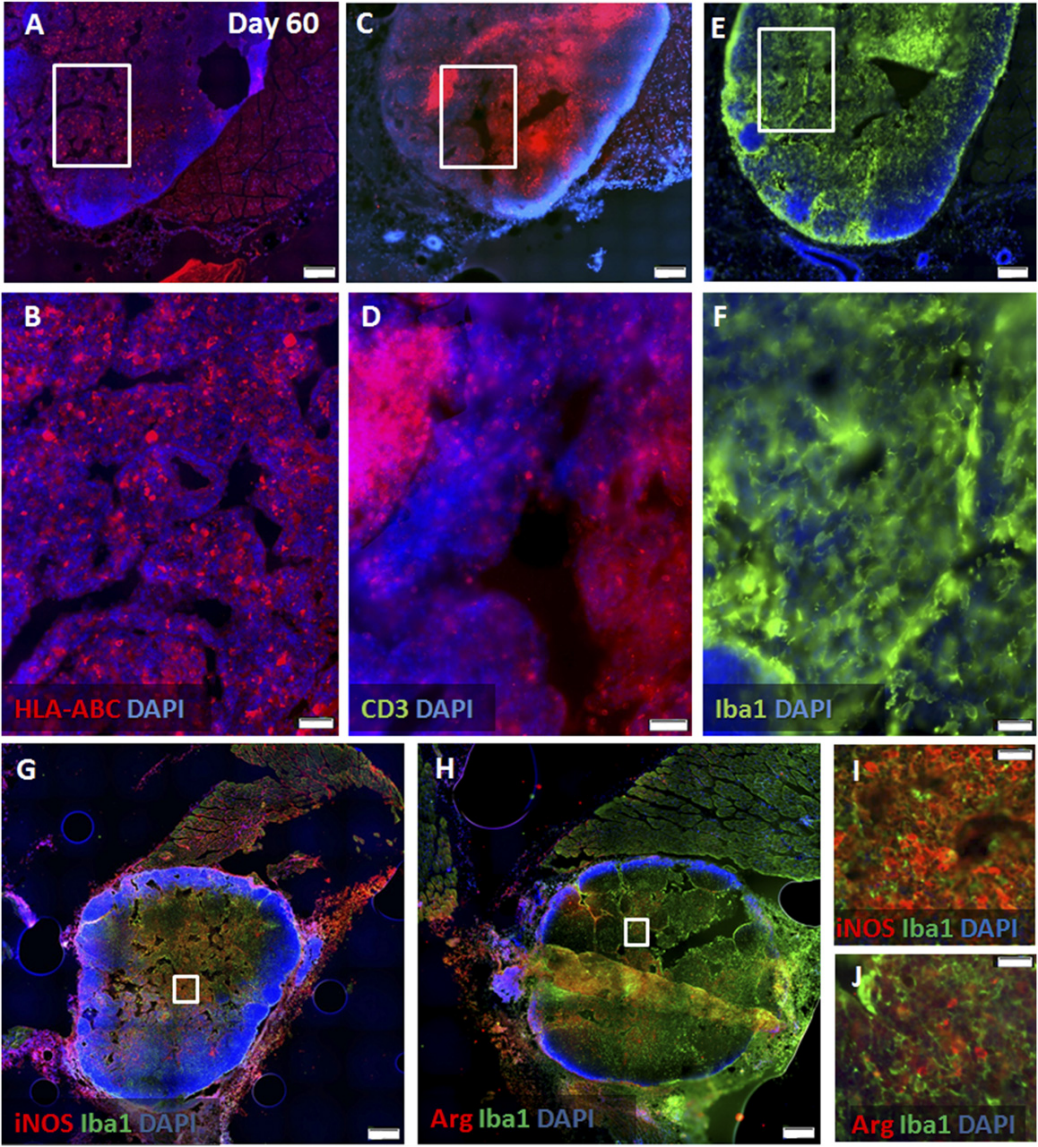
Long‐term survival of hPSCs in muscles along with an aggravated immune response. At 60 days after intramuscular implantation, numerous hPSCs were still detected in the recipient muscles (*n* = 20) by HLA‐ABC immunostaining **(A, B)**. A further increase in the severity of the local immune response, with formation of ectopic lymph node‐like structures was evident in proximity to the graft, as shown by immunostaining for CD3+ T cells **(C, D)** and Iba1+ macrophages **(E, F)**. Characterization of macrophage polarization showed marked domination of classic iNOS+ (M1) macrophages **(G, I)** versus alternative dispersed Arg+ (M2) macrophages **(H, J)**. Insets in **(A, C, E, G, H)** are magnified in **(B, D, F, I, J)**, respectively. Scale bars = 500 µm **(A, C, E, G, H)** and 50 µm **(B, D, F, I, J)**. Abbreviations: Arg, arginase; DAPI, 4′,6‐diamidino‐2‐phenylindole; HLA, human leukocyte antigen; iNOS, inducible nitric oxide synthase.

To examine the therapeutic potential of systemic hPSC delivery, we implanted the cells into muscles. As future clinical translation of hPSC therapy will require the use of allogeneic‐implanted cells, we first examined the survival of xenogeneic mismatched hPSC donor cells in the muscle. The importance of long‐term survival of implanted cells is highlighted by the chronic nature of the EAE model and human MS. Implanted hPSCs were detected within the injected muscles on days 28 (Fig. 3A, 3D) and 60 after implantation (Fig. 4A, 4D). The surviving cells elicited a massive immune response involving both T cells and macrophages in their site of implantation (Figs. 3, 4). The intensity of this response increased with time. On day 60, the formation of ectopic lymph node‐like structures was evident in the muscles of the implanted animals, suggesting an ongoing graft‐elicited immune response. Furthermore, staining for markers of macrophage polarization toward classic versus an alternative phenotype showed marked domination of massive iNOS+ (M1) macrophages (Fig. 4G, 4I) and a small number of dispersed arginase‐positive (M2) macrophages (Fig. 4H, 4J). These findings suggest that the inflammatory response is toward graft rejection.

### Intramuscular hPSC Injection After the Onset of Clinical Signs Does Not Affect the Course of EAE

**Figure 5:**
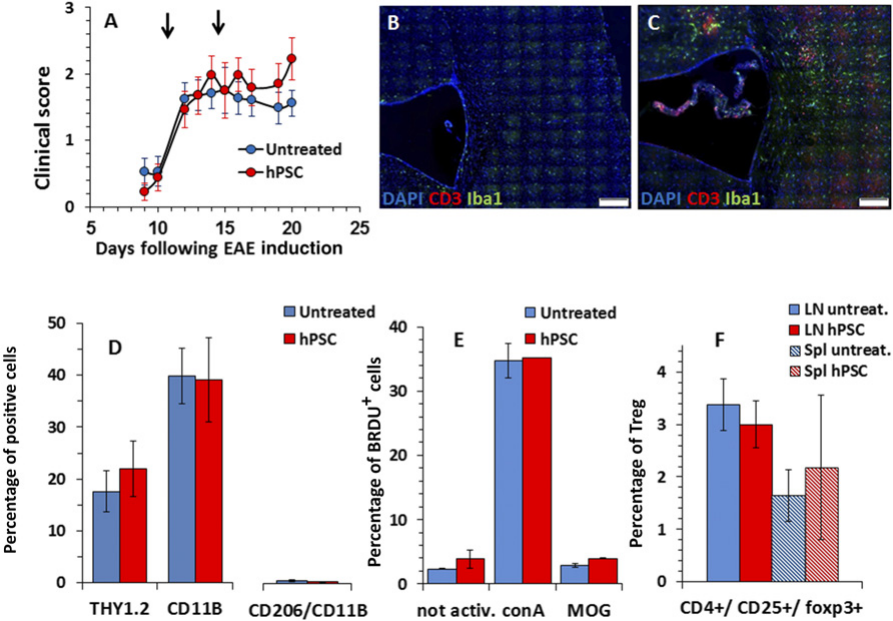
Intramuscular hPSC injection after the onset of clinical signs did not affect the course of EAE; 2 × 10^6^ of hPSCs were injected i.m. on days 11 and 15 (arrows) after EAE induction. **(A):** No difference was observed in disease severity between the untreated control and implanted mice (*n* = 20, each group, **A**). Immunofluorescent staining of brain section for CD3+ T cells and Iba1+ microglia/macrophages showed a certain increase in inflammatory processes in the brains of implanted mice **(C)** compared with untreated mice **(B)**. The immune cells were extracted from the brains and spinal cords of i.m. hPSC‐treated and untreated mice (*n* = 6, per group) and characterized by fluorescence‐activated cell sorting analysis. No difference was found in the fraction or polarization of CD11b+ microglia/macrophages or Thy1.2+ expressing T cells between the two experimental groups **(D)**. To evaluate whether i.m. hPSC delivery induces any systemic immunomodulatory effects, we examined the proliferative response of lymph node cells in vitro on day 22 after EAE induction. ConA induced a similar proliferation of LNCs in the implanted and control groups (*n* = 6 per group, **E**). At this time point, myelin oligodendrocyte glycoprotein peptide corresponding to amino acids 35–55 induced only a borderline proliferative response **(E)**. No difference was observed between the fractions of LN‐ and Spl‐derived regulatory T cells in the two groups **(F)**. Scale bars = 100 µm **(B, C)**. Abbreviations: conA, concanavalin A; DAPI, 4′,6‐diamidino‐2‐phenylindole; EAE, experimental autoimmune encephalomyelitis; hPSC, human placental stromal cell; LN, lymph node; LNCs, lymph node cells; MOG, myelin oligodendrocyte glycoprotein; not activ., not activated; Spl, spleen; Treg, regulatory T cells; untreat., untreated.

The clinical course of EAE in the i.m. hPSC‐injected mice was compared with that of the control mice. Two doses of 2 × 10^6^ hPSCs were injected on days 11 and 15 after EAE induction. This timing was chosen to represent the clinical situation of implantation after the onset of the disease. No significant difference was observed in disease severity between the control and hPSC‐implanted mice (Fig. 5A). Immunofluorescence staining for CD3+ T cells and Iba1+ microglia/macrophages showed comparable inflammatory processes in the brains of the control and i.m.‐implanted mice (Fig. 5B, 5C). We extracted and characterized immune cells from the brains and spinal cords of the implanted and control mice. FACS analysis of CNS extracted immune cells showed no difference in the amount and polarization of CD11b+ microglia/macrophages or Thy1.2+ T cells between the two experimental groups (Fig. 5D). To evaluate whether i.m. implantation induced any systemic immunomodulatory effects, we examined the proliferative response of lymph node cells in vitro. ConA induced similar proliferation of LNCs in the implanted and control groups (Fig. 5E). At this time point, MOG35‐55 peptide induced only a borderline proliferative response (Fig. 5E). However, no difference was found in the response of lymph node cells derived from hPSC‐implanted versus control mice (Fig. 5E). We further quantified the fraction of lymph node‐ and spleen‐derived regulatory T cells. No difference was observed between the two groups (Fig. 5F). In summary, these findings showed that i.m. injection of hPSCs after the onset of clinical signs is not beneficial in attenuating EAE.

### Intramuscular hPSC Injection Before EAE Induction Delays Disease Onset and Attenuates Relapse

**Figure 6:**
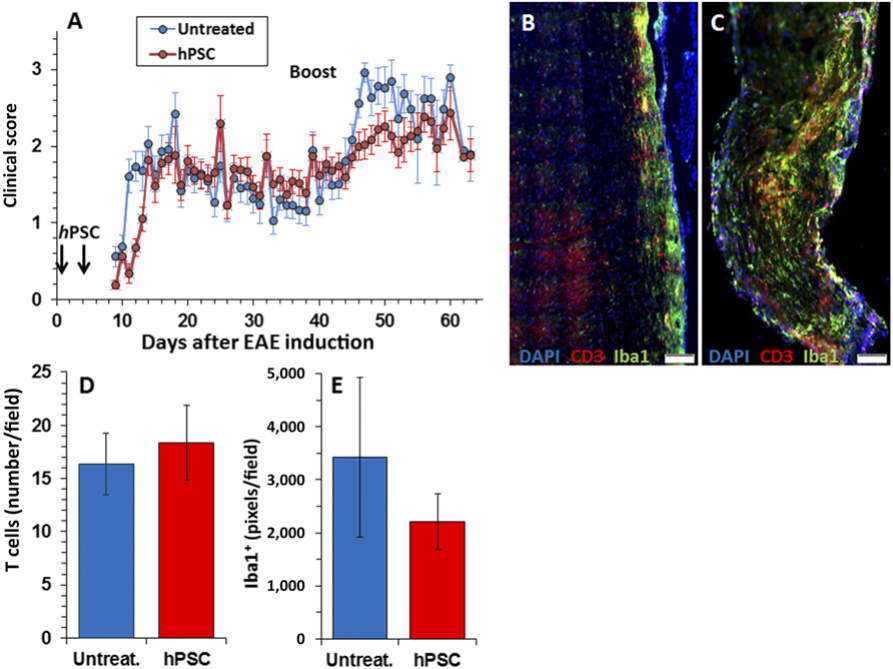
Intramuscular hPSC injection before EAE induction delayed disease onset and attenuated relapse; 2 × 10^6^ hPSCs were injected i.m. on days 1 and 5 after EAE induction before the onset of clinical signs. The clinical scores of all mice participating in this project (*n* = 42 implanted and 45 control mice, part of which were sacrificed at different time points for pathological and immunological studies) were analyzed. **(A):** A mild, but statistically significant, postponement of disease onset by 2 days was observed in i.m. implanted mice (*p* = .026) followed by a comparable disease course. In the postacute phase, no significant difference was found between the groups. To evaluate whether i.m. hPSC treatment exerted long‐term therapeutic effects, a relapse was induced on day 40. Relapse was observed in 85% of the control versus 54% of the hPSC‐treated mice (*p* < .05). The intensity of the relapse was 2.32 ± 1 in the control group versus 1.51 ± 0.43 in the hPSC‐injected mice (*p* < .0005). By day 60, these differences were annulled, and the two experimental groups were identical clinically. Quantification of the inflammatory response in the spinal cord at this time showed no difference in the number of CD3+ T cells, with a mild nonsignificant decline in Iba1+ macrophage/microglia in the hPSC‐treated (*n* = 16; **C–E**) versus control (*n* = 13; **B, D, E**) mice. Scale bars = 100 µm **(B, C)**. Abbreviations: DAPI, 4′,6‐diamidino‐2‐phenylindole; EAE, experimental autoimmune encephalomyelitis; hPSC, human placental stromal cell; Untreat., untreated.

To examine whether systemic delivery of hPSCs exerts any clinical preventive therapeutic effects, we implanted the cells i.m. before the onset of clinical signs of EAE. Two consecutive i.m. injections of hPSC were performed at the first and fifth day after EAE induction. A mild, nearly significant, postponement of disease onset by 2 days was observed (*p* = .08; Fig. 6A), followed by a comparable disease course. In the postacute phase, a nonsignificant trend was seen of better recovery in the control mice. However, the overall course of the disease was similar.

**Figure 7:**
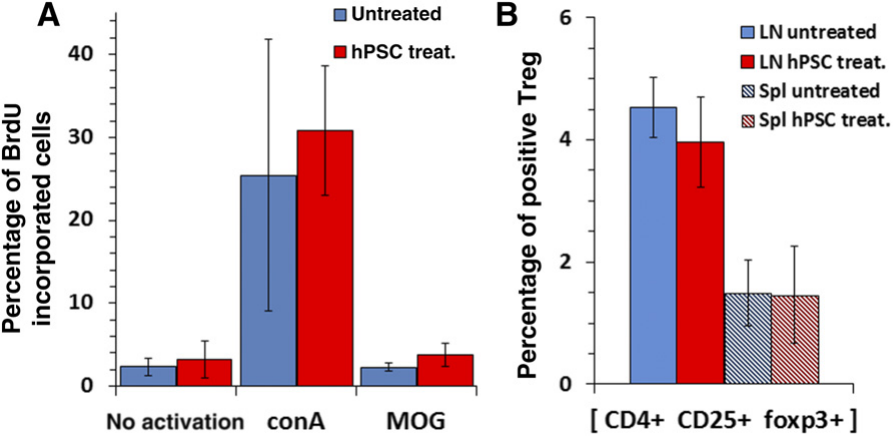
Intramuscular hPSC implantation before EAE induction did not reduce systemic LNC response. To assess whether hPSC i.m. injection before the onset of clinical signs attenuated the systemic immune response, a proliferative BrdU incorporation assay was conducted on LNCs excised on day 10 after EAE induction. Both ConA and MOG peptide stimulation caused a similar proliferative response in the control and hPSC‐injected mice **(A)**. Also, no difference was detected in the fraction of CD4+, CD25+, Fox3+ regulatory T cells from the lymph nodes and spleen of hPSC‐treated and control groups (*n* = 6 per group). Abbreviations: BrdU, bromodeoxyuridine; conA, concanavalin A; hPSC, human placental stromal cell; LN, lymph node; MOG, myelin oligodendrocyte glycoprotein; Spl, spleen; Treg, regulatory T cells; treat., treated.

To evaluate whether i.m. hPSC implantation exerts long‐term therapeutic effects, a relapse was actively induced on day 40. Occurrence of clinical relapse was defined as deterioration by at least one score. A relapse was observed in 85% of the control mice versus 54% in the hPSC‐injected mice (chi‐square *p* < .05). The intensity of the relapse was 2.32 ± 1 in the control group versus 1.51 ± 0.43 in the hPSC‐injected mice (*p* = .0005; Fig. 6A). By day 60, the clinical severity of the disease was identical. Quantification of the inflammatory response in the spinal cord at this time point showed no difference in the numbers of CD3+ T cells (Fig. 6B–6D) and a mild nonsignificant decline in Iba1+ macrophage/microglia in the implanted mice (Fig. 6B, 6C, 6E). Given the mild inhibition of EAE in vivo, we hypothesized that hPSC implantation might reduce T‐cell encephalitogenicity. We used the BrdU incorporation assay to test the in vitro proliferation of LNCs obtained 7 days after MOG35‐55 immunization from either control or hPSC‐implanted mice. No difference was observed in LNC proliferation after either ConA or MOG stimulation (Fig. 7A). In addition, no difference was detected in the fraction of regulatory T cells in the lymph nodes and spleen (Fig. 7B). In summary, a mild clinical beneficial effect was observed after i.m. implantation of hPSCs before disease onset but without a measurable effect on the peripheral immune cells.

## Discussion

Adult stromal cell therapies for MS is an emerging approach that have already reached the stage of clinical trials. The choice of the optimal cell platform and the most useful route of implantation are key issues still under debate. hPSCs were previously shown to obtain superior expansion properties than the extensively studied bone marrow‐derived MSCs [21] and their therapeutic potential after implantation in models of neurological diseases. Given their potential advantages, we tested the applicability of peripheral delivery of hPSCs for systemic therapy in EAE. First, we verified their immunomodulatory properties in inhibiting EAE by intracranial implantation. Then, we assessed the effects of i.m. hPSC implantation on EAE. We found that muscular hPSC grafts induced only a mild, nonsufficient effect on the clinical course of the disease, and only if implanted before the onset of clinical signs, but did elicit a vigorous local immune response.

MS is an immune‐mediated disease targeting the CNS. Therefore, reducing the encephalitogenicity of autoreactive lymphocytes in the periphery might drastically attenuate disease progression. Indeed, i.v. injection of neural precursor cells [2] and bone marrow‐derived MSCs [2, 4] inhibited the encephalitogenicity of autoreactive lymphocytes in the lymph nodes, before their egress and entry to the CNS, with consequent prevention of EAE. Similarly, intraperitoneal implantation of MSCs attenuated EAE via a systemic immunomodulatory effect without infiltrating the CNS [22, 23]. Intramuscular implantation of placental stromal cells was shown to improve recovery in an ischemia‐induced limb injury model via immunomodulation [15] and to mitigate the lethal effects of radiation [16]. Our study showed that a mixture of maternal and fetal hPSCs were more effective than maternal cells alone [16]. Therefore, in the present study, examining this route of cell delivery in an MS model, we used a well‐defined mixture of ∼80% fetal and ∼20% maternal cells.

The immunomodulatory effect of hPSCs seemed to be at least partially mediated via an increased number of regulatory T cells and decreased lymphocyte proliferation due to secretion of interleukin‐10 and transforming growth factor‐β [24]. Therefore, we examined the effect of intramuscular injection of hPSCs in an EAE mouse model as an alternative systemic route of delivery, which also enables the doubling of implanted cell number in repeated injections with no apparent adverse effects. Although our findings showed that hPSCs were highly effective when delivered directly to the CNS, they exhibited only a marginal clinical benefit after i.m. administration, with no difference in the number of systemic regulatory T cells between the nontreated and hPSC‐implanted mice. In addition, no difference was found in the proliferation of isolated lymph node cells in response to ConA or MOG35‐55 peptide. The marginal clinical benefit of i.m.‐implanted hPSC might have resulted from the lack of their activation in the muscle. In a parallel study on hPSC influence on bone marrow regeneration, we demonstrated that i.m.‐delivered hPSCs released vast amounts of protective cytokines only in response to disease‐related stress compared with minimal secretion in nonchallenged animals (unpublished data). One could speculate that hPSCs are activated to a functional state by the local CNS inflammatory signals during EAE, which might not reach remote sites, such as muscle tissue.

The discrepancy between the significant beneficial effects after intracranial delivery compared with the marginal benefit following systemic delivery underscores the importance of the route of cell delivery. Several studies support the notion that hPSCs induce their beneficial effects locally, as bystanders, and therefore should be delivered directly to the target organ of disease [25, 26, 27, 28]. In agreement with our findings, ICV implantation of hPSCs attenuated EAE [8] and reduced the ischemic lesion size after middle cerebral artery occlusion, accompanied by a reduction of the microglial response [29]. Moreover, we found that systemic implantation after the onset of clinical signs, when the inflammatory process has already infiltrated the CNS, had no detectable beneficial effect. This is especially important when addressing the issue of clinical translation in secondary progressive MS patients, because at this stage the disease is compartmentalized within the CNS with the formation of ectopic lymph node‐like structures [30], and systemic immunomodulatory therapies are no longer effective [31].

Of note was that i.m. implantation of hPSCs induced a massive local immune response, with the formation of ectopic lymph node‐like structures, although the cells were not fully rejected by this reaction, as they were detected both 28 and 60 days after their i.m. injection. This might imply that the injected hPSCs are relatively resistant to the immune rejection. The appearance of tertiary lymphoid structures in proximity to grafts with domination of iNOS+ macrophages is considered a good correlate for ongoing rejection.

## Conclusion

Intramuscular hPSC implantation had a marginal effect in preventing EAE and no effect in treating established disease. Our results support the rationale of delivering potent immunomodulatory adult stromal cells with therapeutic and trophic properties directly to the CNS in multiple sclerosis.

## Author Contributions

I. Shapira: collection and/or assembly of data, provision of study material or patients, data analysis and interpretation; N.F.: conception and design, collection and/or assembly of data, manuscript writing; M.T., E.V., and C.M.: preparation and provision of study material or patients; I. Stav: data analysis and interpretation; T.B.‐H.: conception and design, manuscript writing, final approval of manuscript; R.G.: conception and design, preparation and provision of study materials or patients, data analysis and interpretation, manuscript writing, final approval of manuscript.

## Disclosure of Potential Conflicts of Interest

T.B.‐H. is a scientific consultant and science advisory board member of Regenera Pharma, Kadimastem, MAPI Pharma, Stem Cell Medicine, and Sipnose and has stock options in Regenera Pharma, Kadimastem, and MAPI Pharma. The other authors indicated no potential conflicts of interest.
